# Application of a new German law as a basis for end-of life decisions in a medical ICU

**DOI:** 10.1186/cc11105

**Published:** 2012-03-20

**Authors:** R Riessen, C Bantlin, M Haap

**Affiliations:** 1 University Hospital Tübingen, Germany

## Introduction

In 2009 a new German law came into effect that clarified issues regarding end-of-life decisions, especially the role of patient autonomy and the importance of a medical indication in the course of treating patients with terminal illness. In this study we analyzed the end-of-life (EOL) policies in our medical ICU with a focus on the practicability of this law.

## Methods

A retrospective analysis of all patients that were treated in the medical ICU of a large German university hospital in 2009 and 2010 and died during their hospital stay.

## Results

During the observation period 3,401 patients were treated in our ICU. The ICU mortality was 15% (*n *= 501), hospital mortality was 19% (*n *= 658). The mean predictive mortality derived from the SAPS 2 score was 29% for all patients (standardized mortality ratio 0.67), deceased patients had a predictive mortality of 56%. Of all deceased, 232 (35%) had received CPR, 170 of those (73%) outside the ICU. Of all patients who died in the hospital, 126 (19%) had received unlimited therapy. Life support was withdrawn in 245 patients (37%) and life support was withheld in 241 patients (36%). In 46 patients (7%) palliative care was instituted right from the beginning of the ICU stay. In 104 cases (16%) the patients themselves made the EOL decision, in 78 cases (12%) an advance directive was present. A legally designated healthcare proxy was involved in 8%. In 541 cases (82%) the relatives were integrated in EOL decisions with the objective of finding a broad consensus; however, in these cases the assessment of the medical indication and the prognosis by the medical team was of particular importance. Cases in which relatives were not involved in EOL decisions were in 76% cases with short unsuccessful maximal therapy, for example CPR (median ICU stay 5 hours). The rate of life support withdrawal was highest (60%) in patients with CNS diseases. We did not experience any serious or unsolvable conflicts with relatives. Involvement of a law court was necessary in none of the cases.

## Conclusion

EOL policies were applied in 81% of our intensive care patients who died during their hospital stay. The new German law regulations served as a practical and realizable basis for EOL policies in our medical ICU.

